# Characterization of children hospitalized with traumatic brain injuries after building falls

**DOI:** 10.1186/s40621-018-0141-3

**Published:** 2018-04-10

**Authors:** Kirsten V. Loftus, Tara Rhine, Shari L. Wade, Wendy J. Pomerantz

**Affiliations:** 1 Division of Emergency Medicine, Cincinnati Children’s Hospital Medical Center, Cincinnati, OH USA; 2 Division of Physical Medicine and Rehabilitation, Cincinnati Children’s Hospital Medical Center, Cincinnati, OH USA

**Keywords:** Fall, Building fall, Traumatic brain injury

## Abstract

**Background:**

Unintentional falls cause a substantial proportion of pediatric traumatic brain injury (TBI), with building falls carrying particularly high risk for morbidity and mortality. The cohort of children sustaining building fall-related TBI has not been well-examined. We sought to characterize children hospitalized with building fall-related TBIs and evaluate if specific factors distinguished these children from children hospitalized with TBI due to other fall mechanisms. We secondarily assessed if TBI severity among children injured due to a building fall varied between children from urban versus non-urban areas.

**Methods:**

This was a secondary analysis of the Pediatric Health Information System (PHIS), an administrative database from pediatric hospitals. We identified children < 15 years old, hospitalized between 2009 and 2014, with an associated TBI-related diagnosis due to a fall as determined by International Classification of Diseases, Clinical Modification, Ninth revision (ICD9-CM) diagnosis codes. Urban versus non-urban status was determined using PHIS-assigned Rural-Urban Commuting Area codes. Injury severity (i.e. Injury Severity Score (ISS) and head Abbreviated Injury Scale (AIS) score) were calculated. Head AIS scores were dichotomized into minor/moderate (1–2) and serious/severe (3–6) for analysis. Frequencies, descriptive statistics, Chi-square analysis, and Mann-Whitney U analysis characterized populations and determined group differences.

**Results:**

The study cohort included 23,813 children, of whom 933 (3.9%) fell from buildings. Within the building fall cohort, 707 (75.8%) resided in urban areas, 619 (66.3%) were male, 513 (55.0%) were white, and 528 (56.6%) had government insurance; the mean age was 3.8 years (SD 2.9). There was a larger proportion of children with serious/severe TBI among those injured from building falls relative to other falls (63.4% vs 53.9%, *p* <  0.01). Among children injured from building falls, those from non-urban areas were more likely to sustain a serious/severe TBI relative to urban children (58.9% vs 53.6%, *p* <  0.01).

**Conclusions:**

Children hospitalized following buildings falls with TBI sustained more severe injuries relative to other fall types. Although a majority of children hospitalized with building fall related-TBIs were from urban areas, those from non-urban areas frequently sustained serious head injuries. Future research should target expanding prevention efforts to include non-urban areas.

## Background

Pediatric traumatic brain injury (TBI) represents a substantial health burden in the United States (US) and is a leading cause of morbidity and mortality in children. (Kuppermann et al., 2009; Taylor et al., 2017; Centers for Disease Control and Prevention, 2016a) The Centers for Disease Control has estimated that each year there are over 15,000 hospitalizations and 1500 deaths related to TBI among children. (Taylor et al., 2017; Centers for Disease Control and Prevention, 2016a) Unintentional falls are the leading cause of pediatric TBI (Centers for Disease Control and Prevention, 2016a; Faul et al., 2010), and falls from buildings carry a particularly high risk for morbidity and mortality relative to other fall mechanisms. (Barlow et al., 1983; Spiegel & Lindaman, 1977; Stone et al., 2000) TBI is one of the most common types of injury sustained when a child falls from a building, reportedly occurring in about one-third of cases. (Vish et al., 2005; Lehman & Schonfeld, 1993; Lallier et al., 1999) Children injured due to building falls constitute an important population who are likely sustaining some of the most severe, potentially preventable, TBIs associated with unintentional falls. However, the epidemiology of children who fall from buildings has not been extensively examined. Identification of potential factors associated with hospitalization for TBI due to building falls could facilitate the generation of hypotheses for further investigation to better understand which populations are at increased risk for sustaining injury and warrant targeted prevention efforts.

In the early 1970s, a community health initiative in New York City, “Children Can’t Fly,” demonstrated drastic reductions in rates of building falls through community education and public policy. (Barlow et al., 1983; Spiegel & Lindaman, 1977; Stone et al., 2000; Smith et al., 1975) Subsequently, this approach to reduction of this preventable cause of pediatric injury has been adopted as a priority across the country. Despite nearly 1 in 5 children in the US living outside of urban cores (US Department of Health and Human Services et al., 2011), little is known about the associated morbidity from building falls in non-urban (i.e. suburban and rural) communities. (Stone et al., 2000; Benoit et al., 2000)

We sought to use a robust pediatric data set to characterize the population of children less than 15 years of age who were hospitalized with a building fall-related TBI and contrast this cohort with children hospitalized with TBI due to other types of fall mechanisms. We hypothesized that children who fell from buildings would sustain more severe TBI than children injured by other types of unintentional falls. Among those children injured due to a building fall, we secondarily compared TBI severity between children from urban versus non-urban areas.

## Methods

### Study design

This study was a secondary database analysis of children hospitalized with a TBI related to a fall. The study was exempt from Institutional Review Board review.

### Data source

We identified patients from the Children’s Hospital Association’s Pediatric Health Information System (PHIS), an administrative database that contains information from US pediatric hospitals. Hospitals within the PHIS are located in urban cores serving every area in the country, including rural areas from all 50 states. (Peltz et al., 2016) PHIS data elements collected include: dates of service, patient demographics, payer status, International Classification of Diseases, Clinical Modification, Ninth revision (ICD9-CM) diagnosis codes, procedural codes, resource utilization (e.g. medications), discharge disposition, hospital characteristics, and ICD9-CM external cause of injury codes (E-codes). (Conway & Keren, 2009; Colvin et al., 2013) E-codes are data that represent the external causes of injury and include designation of mechanism of injury and intent (e.g. unintentional). (Centers for Disease Control and Prevention, 2016b) De-identified data in this database undergo reliability and validity checks prior to inclusion. (Conway & Keren, 2009; Colvin et al., 2013) Data were obtained from 40 of the 43 PHIS hospitals that submitted data during the study period. Two hospitals were excluded given inconsistent data submission and one hospital removed their data prior to our study.

### Inclusion and exclusion criteria

Our study cohort consisted of children less than 15 years of age admitted to a PHIS hospital between January 1, 2009 and December 31, 2014 with a TBI due to an unintentional fall. Admission data by year for each hospital was reviewed, and only data for hospitals that consistently submitted E-codes during the study period were included in this analysis, resulting in the exclusion of data from five hospitals. We identified children with a TBI-related diagnosis code as previously described using ICD9-CM diagnosis codes: 800.0–801.9, 803.0–804.9, 850–854.1, and 959.01. (Services UDoHaH, 1989) We then refined our cohort to include children with a specific set of E-codes which designate an unintentional fall (E800-E888) as categorized by the Centers for Disease Control. (Centers for Disease Control and Prevention, 2016b) Since the PHIS only contains data from pediatric hospitals, we limited our population to children < 15 years of age to optimize adequate representation of children hospitalized with TBI due to a building fall, as many children ≥ 15 years of age are admitted to non-pediatric hospitals for trauma-related injuries and not transferred to pediatric hospitals for definitive care. (Walther et al., 2016; Webman et al., 2016)

### Data collection

A standard data set containing individual and hospital-level data was extracted from the PHIS database. Demographic data included: age, race [categorized as white, black, and other (Asian, Pacific Islander, American Indian, and other)], gender, ethnicity (Hispanic or Latino, not Hispanic or Latino, and unknown), and payer type (government, non-government, self-pay, and unknown). Urban versus non-urban was determined using Rural-Urban Commuting Area (RUCA) codes assigned to each encounter by PHIS. This code identifies the type of community based on the patient’s zip code of residence (and is independent of hospital zip code) using US Census data (e.g. population density, urbanization). (Hart et al., 2005) Based on previous models, RUCA codes were categorized into: large urban core (1), small suburban area (2), large rural town (3), or small rural town/isolated rural area (4). (Peltz et al., 2016) Hospital data included geographic region and trauma level designation. Geographic regions defined by PHIS were Northeast, Central, South, and West. Trauma level accreditation was divided into two categories: 1) level 1 trauma hospitals (corresponding to the highest level of trauma resource utilization) as certified by the state or the American College of Surgeons (ACS), and 2) all other accredited (levels 2 and 3) and non-accredited hospitals.

To assess injury severity, we identified injury and treatment data elements from the PHIS, including type of TBI sustained (e.g. intracranial hemorrhage, skull fracture), operating room (OR) charges, admission to an intensive care unit (ICU), hospital length of stay (LOS), need for mechanical ventilation, hospital billed charges, and in-hospital patient mortality. Injury severity was also qualified using the total Injury Severity Score (ISS) and the head Abbreviated Injury Scale (AIS) score, which are two metrics that assess injury severity in pediatric trauma. These scores were calculated from PHIS data elements using ICDMAP-90 injury coding software (Services UDoHaH, 1989; Mackenzie & Sacco, 1997), which approximates these scores using administrative coding data and has been validated in the pediatric trauma literature. (Durbin et al., 2001; Fleischman et al., 2017) The total ISS was used to determine total injury burden. To calculate the total ISS, an AIS score is assigned to six different body regions (head/neck, face, chest, abdomen, extremity, and external) and then the squares of the three most severe AIS scores are summed together, with a total ISS range of 0–75. (Baker et al., 1974) For clinical interpretation, the ISS can be dichotomized at a cut point of ≥ 15 to denote more severe injury burden. (Bennett et al., 2012; Bowman et al., 2008; Aiolfi et al., 2017) The head AIS score, a sub-category of the total ISS, was used to denote TBI severity. Scores range from one (mild) to six (not survivable), with scores of 1–2 representing minor to moderate injury (e.g. concussion, simple skull fracture) and scores of 3–6 representing serious/severe injury (e.g. subarachnoid hemorrhage, depressed skull fracture). (Bennett et al., 2012; Bowman et al., 2008; Aiolfi et al., 2017)

### Statistical analysis

Frequencies and descriptive statistics were used to characterize the populations. When appropriate, Chi-square and Mann-Whitney U analyses determined group differences. We compared group differences between the cohort that fell from buildings and the cohort injured due to all other types of falls. Within the cohort that fell from buildings, we compared group differences between urban and non-urban populations, as determined by RUCA code. The “non-urban” population included data from children with RUCA codes 2–4. The decision to combine group data for analyses was determined post hoc, as there were no significant demographic or injury-related differences among them. All data were analyzed using IBM^®^ SPSS^®^ (Version 24.0.0.0, 2016) software.

## Results

### Demographics

**Fig. 1:**
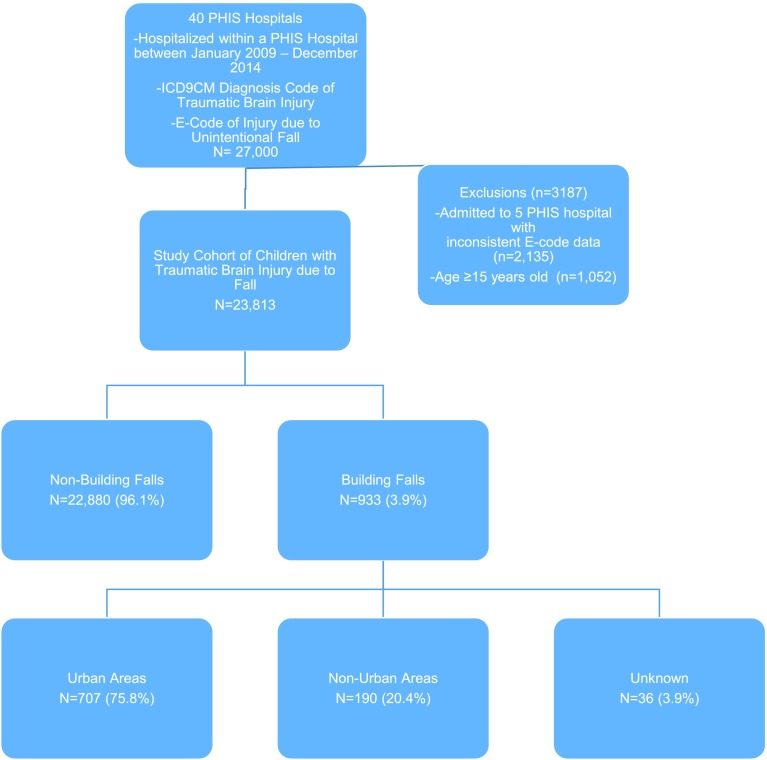
Flow Sheet of Included and Excluded Patients. PHIS = Pediatric Health Information System; ICD9CM = International Classification of Diseases, Clinical Modification, Ninth Revision; E-Code = External Cause of Injury Code

Among 35 PHIS hospitals, we identified 23,813 children under the age of 15 years who were hospitalized with a TBI due to an unintentional fall, of whom 933 (3.9%) fell from buildings. Of those who fell from buildings, 707 were from urban areas, 190 were from non-urban areas, and 36 children were missing a RUCA code. Figure 1 details those included and excluded from the study cohort. The demographics of the study population and group comparisons between children injured due to building falls versus all other types of falls are in Table 1. A majority of patients in the building fall cohort were male (66.3%), with a mean age of 3.8 years old. Race and payer type differed significantly between children who fell from buildings and children injured due to other types of falls, with a larger proportion of non-white children (22.0% vs 14.7%, *p* <  0.01) and a smaller proportion of children with private insurance coverage (34.1% vs 41.0%, *p* <  0.01) in the building-fall cohort.

**Table 1:** Demographics of Children Hospitalized from 2009 to 2014 with Traumatic Brain Injury Due to Unintentional Falls

Demographics	Building Falls (*n* = 933)	All Other Types of Falls (*n* = 22,880)	*p*-value* (Building vs Other)	Urban Building Falls (*n* = 707)	Non-urban Building Falls^a^ (*n* = 190)	p-value* (Urban vs Non-urban)
Gender (%)						
Male	619 (66.3)	13,484 (58.9)	< 0.01	466 (65.9)	127 (66.8)	NS
Female	310 (33.2)	9357 (40.9)		237 (33.5)	63 (33.2)	
Unknown	4 (< 0.5)	39 (0.2)		4 (0.6)	0	
Mean age in years (SD)	3.79 (2.9)	3.10 (3.9)	< 0.01	3.56 (2.8)	4.49 (3.4)	< 0.01
Race (%)						
White	513 (55.0)	15,265 (66.7)	< 0.01	338 (47.8)	149 (78.4)	< 0.01
Black	205 (22.0)	3358 (14.7)		193 (27.3)	9 (4.7)	
Other	215 (23.0)	4257 (18.6)		176 (24.9)	32 (16.8)	
Ethnicity (%)						
Hispanic/Latino	152 (16.3)	3690 (16.1)	NS	130 (18.4)	18 (9.5)	< 0.01
Not Hispanic/Latino	657 (70.4)	16,044 (70.1)		486 (68.7)	140 (73.7)	
Unknown	124 (13.3)	3146 (13.8)		91 (12.9)	32 (16.8)	
Payer type (%)						
Government	528 (56.6)	11,962 (52.3)	< 0.01	433 (61.2)	85 (44.7)	< 0.01
Non-government	318 (34.1)	9390 (41.0)		225 (31.8)	69 (36.3)	
Self-pay	70 (7.6)	1250 (5.5)		37 (5.3)	31 (16.8)	
Unknown	17 (1.8)	257 (1.1)		12 (1.7)	5 (2.6)	
RUCA Code						
Large Urban Core	707 (75.8)	16,986 (74.2)	NS	NA	NA	NA
Small Suburban Area	76 (8.1)	2110 (9.2)				
Large Rural Town	53 (5.7)	1582 (6.9)				
Small Rural Town/	61 (6.5)	1472 (6.4)				
Isolated Rural Area						
Unknown	36 (3.9)	730 (3.2)				

Legend: *SD* standard deviation, *NS* not significant, *NA* not applicable, *RUCA* rural-urban commuting area

*p -values obtained using Chi-square or Mann-Whitney U tests to evaluate for group differences

^a^Reflects children from Small Suburban Areas, Large Rural Towns, or Small Rural Town/Isolated Rural Areas

The majority (75.8%) of children who fell from buildings resided in urban areas. Urban versus non-urban comparisons among children who fell from buildings revealed significant differences with regards to race, ethnicity, and payer type. Those residing in urban areas were less likely to be white (47.8% vs 78.4%, *p* <  0.01), more likely to be Hispanic (18.4% vs 9.5%, *p* <  0.01), and more likely to have government insurance (61.2% vs 44.7%, *p* <  0.01), compared to children from non-urban areas.

### Injury and treatment characteristics

**Fig. 2:**
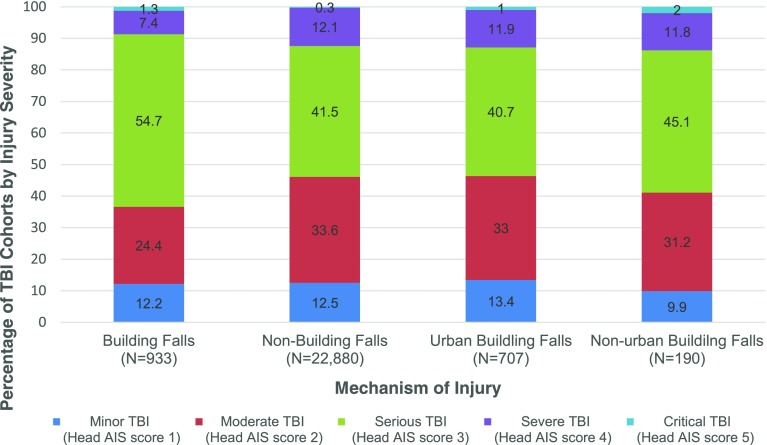
Traumatic Brain Injury Severity* of Children Hospitalized Due to Unintentional Falls from 2009 to 2014. *Traumatic brain injury severity was identified using the head abbreviated injury scale. TBI = traumatic brain injury; AIS = abbreviated injury scale

Figure 2 illustrates the distribution of TBI severity based on the head AIS score within our cohort by type of fall. There was a significantly larger proportion of children with serious/severe TBI (i.e. head AIS score of 3–6) among those injured due to building falls, relative to other types of falls (63.4% vs 53.9%, *p* <  0.01). Among children injured due to buildings falls, children from non-urban areas were significantly more likely to sustain serious/severe TBI relative to urban children (58.9% vs 53.6%, *p* <  0.01).

The diagnoses of isolated concussion and isolated skull fracture were identified at similar rates (approximately 15% and 30%, respectively) among children who fell from buildings and all other types of unintentional falls. Children injured due to a building fall were more likely to sustain skull fracture with extra-axial hemorrhage (30.1% vs 21.7%, *p* <  0.001) and less likely to sustain isolated intracranial injury (5.6% vs 14.4%, *p* < 0.001) relative to children injured from other types of falls. Within the building fall cohort, the diagnoses of isolated skull fracture and isolated intracranial injury were identified at similar rates (approximately 30% and 5%, respectively) among children injured in urban and non-urban communities. Children injured in a non-urban area were more likely to sustain skull fracture with extra-axial hemorrhage (36.8% vs 28.9%, *p* = 0.03) and less likely to sustain isolated concussion (8.9% vs 16.1%, *p* = 0.01) relative to children injured from urban areas.

The distribution of ISSs was similar in the building fall and other types of falls cohorts, both having a median ISS of 9 and an interquartile range (IQR) of 4–9. However, children injured due to a building fall were significantly more likely to have sustained an ISS of ≥15 relative to children injured due to other types of falls (18.9% vs 12.9%, *p* < 0.001). There was no difference in the proportion of children from urban (13.0%) versus non-urban (13.4%) environments who sustained ISS ≥ 15.

The injury and hospital care characteristics of the study population are in Table 2. Children hospitalized with a building fall-related TBI were significantly more likely to require an operation, an ICU admission, and mechanical ventilation compared to children sustaining other types of falls. Among children who required an operation, 75% had a serious/severe head AIS score and 66% had an ISS < 15. Although infrequent, in-hospital mortality was more common in the building fall cohort (1.0% vs 0.3%, *p* < 0.01). There were no statistically significant group differences between children from urban versus non-urban areas concerning treatment factors or in-hospital mortality. Most children in our study were admitted to a hospital with level 1 trauma accreditation in the southern region of the US.

**Table 2:** Injury and Hospital Care Characteristics of Children Hospitalized from 2009 to 2014 with Traumatic Brain Injury Due to Unintentional Falls

	Building Falls (n = 933)	All Other Types of Falls (n = 22,880)	p-value* (Building vs Other)	Urban Building Falls (n = 707)	Non-urban Building Falls^b^ (n = 190)	p-value* (Urban vs Non-urban)
Serious/Severe Head Injury^a^ (%)	591 (63.4)	12,332 (53.9)	< 0.01	379 (53.6)	112 (58.9)	< 0.01
Serious/Severe Overall Injury^a^ (%)	176 (18.9)	2957 (12.9)	< 0.001	2294 (13.0)	719 (13.4)	NS
Intensive Care Unit Admission (%)	445 (47.8)	4771 (20.9)	< 0.01	334 (47.2)	85 (44.7)	NS
Operating Room Admission (%)	114 (12.2)	1201 (5.2)	< 0.01	87 (12.3)	25 (13.2)	NS
Mechanical Ventilation (%)	127 (13.6)	725 (3.2)	< 0.01	97 (13.7)	25 (13.2)	NS
Mean LOS in Days (SD)	3.28 (6.6)	1.75 (3.2)	< 0.01	3.2 (6.2)	3.4 (7.6)	NS
Mean Billed Charges (SD)	$38,473 (89,234)	$16,016 (39,730)	< 0.01	$38,633 (89,060)	$35,970 (70,098)	NS
Hospital Region (%)						
Midwest	198 (21.2)	4847 (21.2)	< 0.001	129 (18.2)	69 (36.3)	< 0.001
Northeast	128 (13.7)	3140 (13.7)		102 (14.4)	25 (13.2)	
South	327 (35.0)	10,355 (45.3)		261 (36.9)	57 (30.0)	
West	280 (30.0)	4538 (19.8)		215 (30.4)	39 (20.5)	
Hospital Level 1 Trauma Designation (%)						
Yes	809 (86.7)	18,995 (83.0)	0.003	613 (86.7)	161 (84.7)	NS
No	124 (13.3)	3885 (17.0)		94 (13.3)	29 (15.3)	

Legend: *LOS* length of stay, *SD* standard deviation

^a^Serious/Severe head injury was identified if a child had a head abbreviated injury scale score of 3–6; Serious/Severe overall injury severity was identified if a child had a total injury severity score of ≥ 15

*p -values obtained using Chi-square or Mann-Whitney U tests to evaluate for group differences

^b^Reflects children from Small Suburban Areas, Large Rural Towns, or Small Rural Town/Isolated Rural Areas

Among children hospitalized with a TBI due a building fall, community-type was unknown for 36 (3.9%) children, so they are not included in the urban or nonurban columns

## Discussion

To our knowledge, this is the first study to provide specific emphasis on children who sustain a TBI due to falls from buildings and examine differences in patient characteristics and injury severity among children in urban compared to non-urban areas. Children hospitalized with TBI due to a building fall sustained significantly more severe injuries relative to children injured due to other types of falls. In our study, these children were more likely to sustain serious/severe TBI and had significantly higher rates of ICU admission and mechanical ventilation when compared to children hospitalized with TBI due to other types of falls. Comparisons of children hospitalized with building fall-related TBIs from urban versus non-urban communities revealed comparable rates of serious/severe TBIs and similar overall injury severity scores.

While unintentional falls are known to be a common mechanism of pediatric injury, our study highlights that falls can contribute to substantial, and sometimes fatal, TBI. Children who fall from buildings are at particularly high risk for more severe TBI relative to other types of falls in both urban and non-urban communities. Mortality in our building fall cohort was infrequent, although similar to the previously reported 0–5% death rate. (Barlow et al., 1983; Stone et al., 2000; Vish et al., 2005; Lehman & Schonfeld, 1993; Lallier et al., 1999; Benoit et al., 2000; Istre et al., 2003) Our study compliments prior research by demonstrating not only increased injury severity, but also increased resource utilization among those children hospitalized with a TBI due to building falls relative to other types of falls. (Barlow et al., 1983; Spiegel & Lindaman, 1977; Vish et al., 2005; Lehman & Schonfeld, 1993; Benoit et al., 2000) Further investigation of children at risk of TBI due to building falls and expansion of current prevention efforts is important in reducing the preventable morbidity and mortality associated with these injuries.

As detailed in Table 2, our study also suggests that building falls in non-urban areas may result in even more severe injuries, possibly related to better preventive efforts and legislation in urban areas or differences in the types of building falls in urban versus non-urban areas. Prior research has not compared TBI severity between children who fall from buildings in urban versus non-urban areas. Although studies have found that urban children are more likely to have higher total ISSs due to multiple injuries relative to children injured in non-urban areas (Stone et al., 2000; Benoit et al., 2000), our findings demonstrated similar overall injury severity among children from urban and non-urban areas. Additionally, we were surprised to identify a significantly higher proportion of children with serious/severe TBI among non-urban children relative to children from urban areas, although the difference between the groups was small. Since our data are from a more recent time period than previous literature, these findings may be reflective of fewer urban children falling from high-rise buildings secondary to sustained public policy and prevention. It is also possible that children in non-urban areas with more severe injuries are more likely to require transfer to an urban, pediatric center, which may bias these results.

When examining resource utilization (e.g. ICU admission, mechanical ventilation), we found no significant group differences between the urban and non-urban cohorts, which is likely due to the large proportion of children with a head AIS of 3. The decision to dichotomize the AIS was made a priori to assist with clinical interpretation of our data, and while this score denotes moderate injury severity, (e.g. cerebral contusion, subarachnoid hemorrhage), it may not always be associated with critical care. Our findings underscore that children who fall from buildings are continuing to sustain significant TBI with substantial resource utilization and cost.

A majority of patients in the building fall cohort were male, which is consistent with existing literature on pediatric patients presenting to an ED after a building fall. (Barlow et al., 1983; Stone et al., 2000; Vish et al., 2005; Lehman & Schonfeld, 1993; Benoit et al., 2000; Istre et al., 2003) The majority of children injured due to building falls have been reported to be ≤ 4 years old. (Barlow et al., 1983; Stone et al., 2000; Vish et al., 2005; Lehman & Schonfeld, 1993; Benoit et al., 2000; Istre et al., 2003) Our findings support this with most of our building and non-building fall cohort being toddler-aged, including both children from urban and non-urban areas. Within our building fall cohort, urban children were more likely to be black, Hispanic/Latino, and have government insurance compared to non-urban children.

There are limitations to consider when interpreting our results. E-codes were not available for every patient in the database, and we excluded those with missing E-codes. We did limit inclusion to only hospitals that consistently submitted E-code data during our study period to avoid selection bias. Additionally, given this is an administrative database, there is the possibility for misclassification bias due to coding errors. When using a large database, statistically significant group differences should always be assessed for clinical significance. Since PHIS is an administrative database, there is no narrative detailing building types (e.g. high-rise building, barn) or fall height in order to characterize differences in urban versus non-urban falls that could further inform prevention efforts. Our study population was limited to patients with a diagnosis of TBI and did not extensively detail additional injuries that patients may have sustained due to falls. Although additional injuries may have contributed to resource utilization, given that most children had a head AIS of 2–3 and an ISS of < 10, it seems likely that the majority of children in this large dataset were in fact hospitalized for their TBI. Our study only looked at urban versus non-urban, so differences among types of non-urban areas could not be fully assessed given small numbers, but we did not identify any significant group differences prior to combining the non-urban data. Due to the dichotomization of urban versus non-urban for data analysis, there is the risk of classification bias. Our study population included only children admitted to the hospital, thereby excluding children with minor injuries who were discharged home and children with more severe injuries who died prior to admission. Although this may affect our cohort’s generalizability, being discharged home after a building fall and dying immediately after a building fall are uncommon. In addition, our data do not include any admissions at non-pediatric centers, which may impact generalizability, especially for children sustaining more minor TBI.

## Conclusions

TBI related to building falls results in significant injury severity and health care utilization, representing a substantial health burden in the US. Although there were more children in urban areas hospitalized with TBI after a building fall, those in non-urban areas frequently sustained serious head injuries. Future prevention efforts regarding this very preventable injury should be expanded to include non-urban areas.

## Abbreviations

ACS: American College of Surgeons
AIS: Abbreviated Injury Scale
E-codes: External cause of injury codes
ICD9-CM: International Classification of Diseases, Clinical Modification, Ninth revision
ICU: Intensive care unit
ISS: Injury Severity Score
LOS: Length of stay
OR: Operating room
PHIS: Pediatric Health Information System
RUCA: Rural-Urban Commuting Area
TBI: Traumatic brain injury
US: United States
